# A new species of the genus *Helcogramma* (Blenniiformes, Tripterygiidae) from Taiwan

**DOI:** 10.3897/zookeys.216.3407

**Published:** 2012-08-21

**Authors:** Min-Chia Chiang, I-Shiung Chen

**Affiliations:** 1 Institute of Marine Biology, National Taiwan Ocean University, Keelung 202, Taiwan, ROC; 2 Center for Marine Bioenvironment and Biotechnology (CMBB), National Taiwan Ocean University, Keelung 202, Taiwan, ROC

**Keywords:** Fish fauna, fish taxonomy, marine fish, new triplefin, Taiwan

## Abstract

A new species of triplefin fish (Blenniiformes: Tripterygiidae), *Helcogramma williamsi*, is described from six specimens collected from southern Taiwan. This species is well distinguished from its congeners by possessing 13 second dorsal-fin spines; third dorsal-fin rays modally 11; anal-fin rays modally 19; pored scales in lateral line 22-24; dentary pore pattern modally 5+1+5; lobate supraorbital cirrus; broad, serrated or palmate nasal cirrus; first dorsal fin lower in height than second; males with yellow mark extending from anterior tip of upper lip to anterior margin of eye and a whitish blue line extending from corner of mouth onto preopercle. Comparisons and a diagnostic key are provided for the species of *Helcogramma* now known from Taiwan: *Helcogramma fuscipectoris*, *Helcogramma inclinata*, *Helcogramma striata*, *Helcogramma trigloides*, and the newly recorded, *Helcogramma rhinoceros*.

## Introduction

The genus *Helcogramma* McCulloch & Waite (1918) contains small to medium sized tripterygiid fishes with rather fusiform bodies. It can be distinguished from other genera of Tripterygiidae by the following combination of features: a single continuous lateral line; first dorsal fin with three spines; anal fin with a single spine; pelvic fin with one hidden spine and two simple rays (Rosenblatt 1960; Fricke 1997). Species of the genus *Helcogramma* share the following characters: lateral line with 12-37 pored scales, curving ventrally from the posttemporal to mid-body and extending to below the second or third dorsal fin or onto caudal peduncle; spine of anal fin usually less than half the length of first ray; the two segmented rays of pelvic fin sometimes joined by membrane for part of their length; dentary canals with 1-7 pores at the symphysis and 2-10 on either side; supraorbital cirrus simple to palmate or absent; nasal cirrus simple to palmate. Body with ctenoid scales; nape usually naked, rarely with a few scales; head, abdomen and pectoral-fin base always naked (Hansen 1986; Shen and Wu 1994; Fricke 1997; Holleman 2007).

Fishes of *Helcogramma* are widely distributed through the Indo-West Pacific and southeastern Atlantic. This genus comprises 37 valid species (not including *Helcogramma* sp. listed in Fricke 2009), of which 13 species were described in the past ten years (Williams and Howe 2003; Holleman 2006, 2007). There are at least seven nominal species of *Helcogramma* that have been recorded from Taiwan (Holleman 1982; Hansen 1986; Williams and McCormick 1990; Shen and Wu 1994; Fricke 1997) inclulding: *Helcogramma fuscipectoris* (Fowler, 1946), *Helcogramma fuscopinna* (Holleman, 1982), *Helcogramma habena* (Williams & McCormick, 1990), *Helcogramma inclinata* (Fowler, 1946), *Helcogramma obtusirostre* (Klunzinger, 1871), *Helcogramma striata* Hansen (1986), and *Helcogramma trigloides* (Bleeker, 1858). *Helcogramma fuscipectoris* specimens collected from the Ryukyu Islands of Japan and Taiwan were considered by Hansen (1986) to be a junior synonym of *Helcogramma obtusirostre*. However, the Japanese *Helcogramma fuscipectoris* was classified by Fricke (1997) as a different species from *Helcogramma obtusirostre*,which occurs only in the Red Sea and Oman (Holleman 2007). Some Taiwanese specimens identified by Holleman (1982) and Hansen (1986) as *Helcogramma fuscopinna* were determined to represent a distinct species and were described by Williams and McCormick (1990) as *Helcogramma habena*.Subsequently, *Helcogramma habena* was considered by Fricke (1997), and confirmed by Williams and Howe (2003), to be a junior synonym of *Helcogramma inclinata*, which previously had been synonymized with *Helcogramma hudsoni*. Thus, only four valid species of the genus *Helcogramma* were known from Taiwan prior to this study.

A new species from southern Taiwan is described in the present paper increasing the total number of recognized valid species of *Helcogramma* to 38. We also report a new locality record for *Helcogramma rhinoceros* Hansen (1986) and redescribe Taiwanese specimens of the species known from Taiwan.

## Materials and methods

All Taiwanese specimens examined in this study were collected from 2006-2010 from coastal waters of Taiwan using either hand-nets in tide pools or while SCUBA diving. Specimens used for morphological studies were preserved in 10% formalin before being transferred into 70% ethanol for long-term preservation. The type specimens of the new species and specimens of congeners examined that were collected from Taiwan have been deposited at the Institute of Marine Biology, National Taiwan Ocean University (NTOU-P), Keelung. Other comparative materials, including types, examined in this study are deposited in the National Museum of Natural History, Smithsonian Institution (USNM), Washington DC.

Counts and measurements follow those given by Holleman and Bogorodsky (2012) and Chiang and Chen (2008). Measurements were made with needle-point calipers under a dissecting microscope and recorded to the nearest 0.1 mm. Proportional measurements given in the text are in relation to standard length (SL), head length (HL) and eye diameter. Meristic abbreviations include A = anal-fin rays and D = dorsal-fin rays. Elements of the three dorsal fins are presented as a formula: number of spines in first dorsal fin, number of spines in second fin, number of segmented rays in third fin. Dentary pore counts are listed as a formula: right dentary + symphyseal + left dentary. Osteological observations were made on cleared and stained specimens and from radiographs. Number of vertebrae represented as precaudal + caudal vertebrae following Holleman (1982).

## Systematics

### Helcogramma williamsi sp. n.

urn:lsid:zoobank.org:act:33C9C3E3-E385-4FC4-8383-80DF75FF20A4

http://species-id.net/wiki/Helcogramma_williamsi

Fig. 1

#### Holotype.

NTOU-P 2012-02-002, male, 27.5 mm SL, Feng-chui-sha, Hengchun Township, Pingtung County, Taiwan, 1-3 m depth, M. C. Chiang and J. H. Huang, 3 June 2008.

#### Paratypes.

5 paratypes were collected with holotype: NTOU-P 2012-02-001, male, 29.0 mm SL; NTOU-P 2012-02-003, male, 25.4 mm SL; NTOU-P 2012-02-004, female, 28.5 mm SL; NTOU-P 2012-02-005, 2 females, 21.1 and 21.3 mm SL.

#### Diagnosis.

The new species can be distinguished from congeners by the following combination of features. Second dorsal-fin spines XIII; third dorsal-fin rays modally 11; anal-fin rays modally 19; lateral line with 22-24 pored scales; pattern of dentary pores modally 5+1+5; nape naked; supraorbital cirrus lobate; nasal cirrus broad, serrated or palmate; first dorsal fin lower in height than second; males with yellow mark from anterior tip of upper lip to anterior margin of eye and a whitish blue line extending from corner of mouth onto preopercle.

#### Description.

D III, XIII-XIV (holotype: XIII), 10-11 (holotype: 11). A I, 19-20 (holotype: 19). Pectoral fin rays 1+8+7, uppermost ray simple, eight middle rays branched, seven lowermost rays simple. Pelvic fin I, 2, rays united by membrane for half the length of shorter ray. Caudal fin rays 2+9+2, two uppermost and lowermost rays simple, nine middle rays branched. Scale rows 36-37. Lateral line with 22-24 (holotype: 24) pored scales, ending below 2^nd^-4^th^ ray of third dorsal fin. Patterns of cephalic sensory canal pores are illustrated in Fig. 2. Dentary with a single symphyseal pore, dentary pore pattern 5-6+1+5-6 (holotype: 5+1+5). Vertebrae 10+26. No free pterygiophore between second and third dorsal fins.

Body moderately elongate and compressed. Head moderately large, dorsal profile triangular. Body covered with ctenoid scales. Head, nape, base of pectoral fin, and abdomen naked; body scales not extending to bases of first and anterior portion of second dorsal fins. Mouth terminal, posteriormost margin of maxilla just reaching vertical through anterior margin of pupil. Eye moderately large and slightly angled dorsally. Supraorbital cirrus lobate, usually with micromelanophores. Anterior nostril a short tube with broad, serrated or palmate nasal cirrus. First dorsal fin lower in height than second in both sexes. Anal fin beginning below vertical through base of 7^th^ or 8^th^ spine of second dorsal fin; pectoral fin large and pointed, posterior tip of longest ray below last spine of second dorsal fin; caudal fin truncate to slightly rounded. Morphometric data are listed in Table 1.

**Figure 1.**
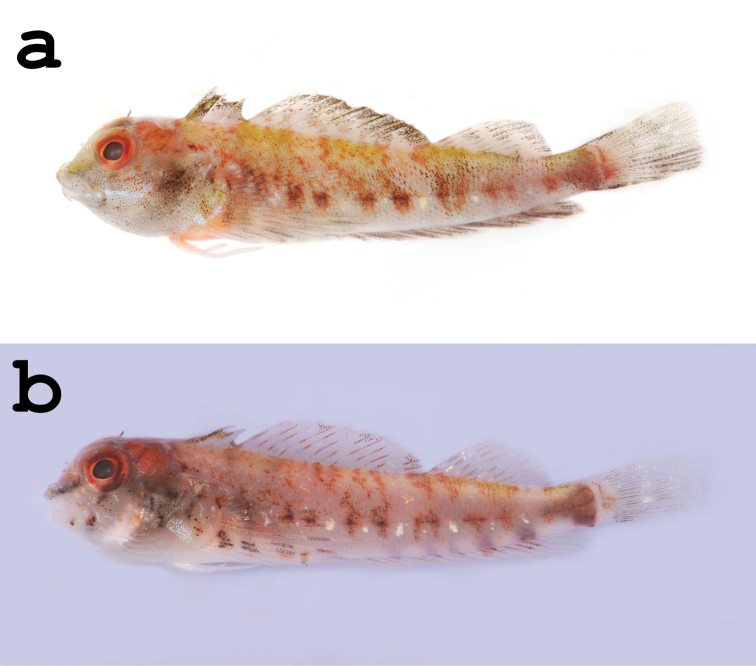
*Helcogramma williamsi* sp. n., Feng-chui-sha, Pingtung, Taiwan **a** Holotype, NTOU-P 2012-02-002, male, 27.5 mm SL **b** Paratype, NTOU-P 2012-02-005, female, 21.3 mm SL.

**Figure 2.**
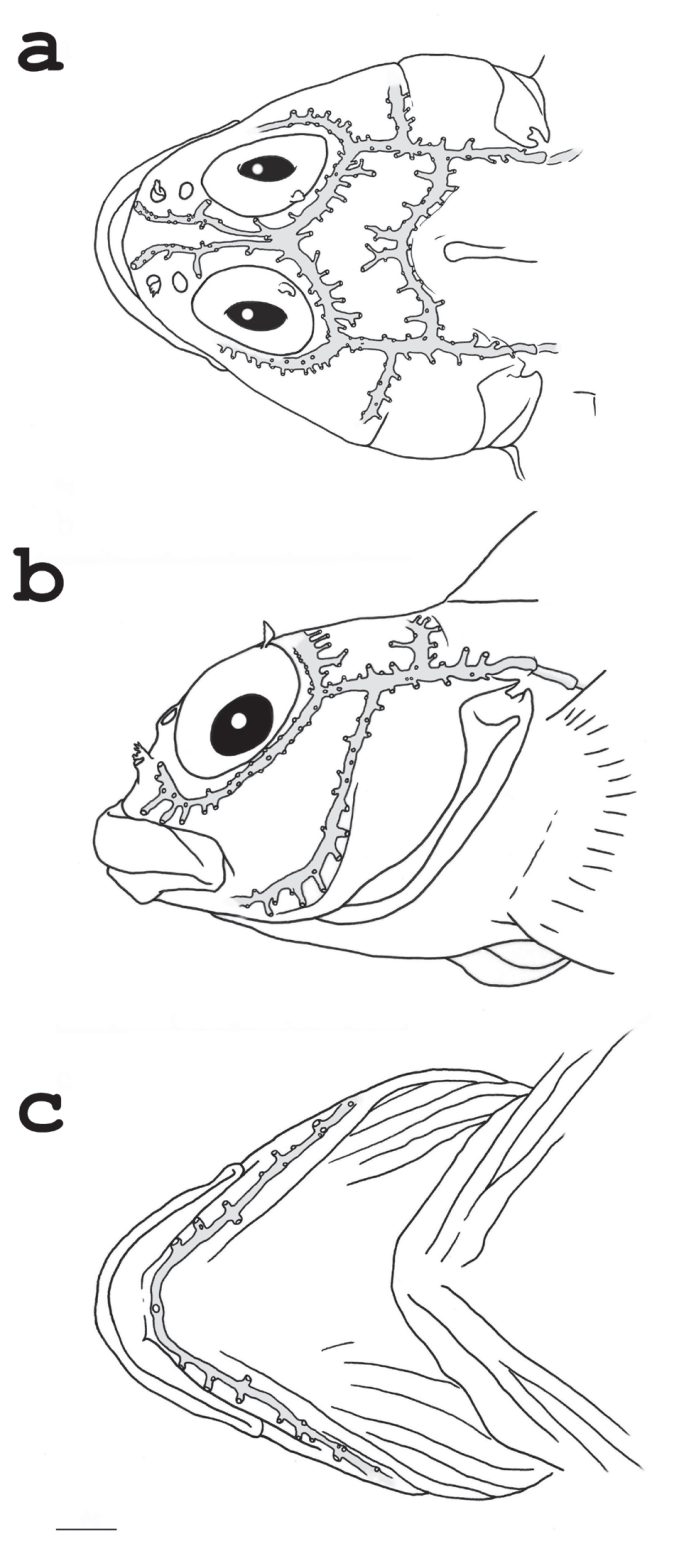
Cephalic sensory canal system of *Helcogramma williamsi* sp. n., holotype, NTOU-P 201202-002, male, 27.5 mm SL **a** Dorsal view **b** Lateral view **c** Ventral view. Canal system indicated in gray. Scale bar = 1 mm.

**Table 1.** Measurements of type specimens of *Helcogramma williamsi*, sp. n.,from Taiwan.

Type status	Holotype	All type specimens (n = 6)			
**Standard length (mm)**	**27.5**	**21.1 – 29.0**	
**In % of standard length**					
Head length	29.1	28.4 – 29.5	( 28.9 )
Body depth of anal fin origin	18.2	16.6 – 18.2	( 17.4 )
Body width of anal fin origin	14.5	12.3 – 14.5	( 13.3 )
Head width in maximum	24.7	23.0 – 24.9	( 24.1 )
Predorsal(1) length	26.2	25.2 – 27.2	( 26.1 )
Predorsal(2) length	37.1	34.0 – 38.5	( 36.0 )
Predorsal(3) length	70.2	68.4 – 71.8	( 70.2 )
Prepectoral-fin length	32.0	32.0 – 33.8	( 33.0 )
Prepelvic-fin length	23.6	22.1 – 26.8	( 23.7 )
Preanal-fin length	48.7	48.7 – 52.6	( 50.4 )
Caudal-peduncle length	10.2	8.5 – 10.2	( 9.4 )
Caudal-peduncle depth	8.7	8.0 – 9.1	( 8.5 )
Pectoral-fin length	33.8	29.0 – 35.7	( 32.8 )
Pelvic-fin length	21.1	20.4 – 21.1	( 20.8 )
Caudal-fin length	20.7	20.3 – 23.0	( 21.2 )
D1 fin base	11.3	9.8 – 11.7	( 10.7 )
D2 fin base	34.9	34.3 – 36.2	( 35.0 )
D3 fin base	19.6	19.3 – 20.4	( 19.9 )
A fin base	43.6	41.1 – 45.3	( 43.1 )
D1 1st spine length	9.8	8.4 – 9.8	( 9.3 )
D2 1st spine length	12.0	11.6 – 13.1	( 12.3 )
**Head length (mm)**	**8.0**	**6.0 – 8.5**	
**In % of head length**					
Head width in maximum	85.0	78.8 – 89.3	( 84.4 )
Eye diameter	30.0	28.0 – 33.3	( 30.5 )
Interorbital width	8.8	8.2 – 9.9	( 9.0 )
Upper-jaw length	43.8	41.0 – 43.8	( 42.4 )
Snout length	35.0	34.4 – 36.5	( 35.0 )
**Eye diameter (mm)**	**2.4**	**2.0 – 2.5**	
**In % of eye diameter**					
Nasal tentacle length	12.5	12.0 – 16.7	14.2
Orbital tentacle length	16.7	15.0 – 20.0	17.9

#### Colouration when fresh.

Males with top of head orange red; lower half of head below eyes, inclusive of lips and branchiostegal membranes, covered with scattered melanophores on pale gray background; opercle heavily spotted and mostly dusky. A whitish blue line extending from posterior flange of maxilla across cheek onto preopercle; faint orange spots below eye and along sides of mouth. Iris orange to red. Snout with iridescent yellow mark, bordered ventroposteriorly by dusky line from anterior margin of eye to anterior tip of upper lip. Body mottled yellow and orange on dorsum; pairs of orange or red, indistinct, slightly angled semi-bars from behind pectoral-fin base to caudal fin, last half-pair forming triangular mark on caudal peduncle. Mid-lateral series of reddish brown blotches, elongating into slender dorsal bars, with intervening white spots. Pectoral-fin base with yellowish white splotch centrally, red and white marks ventrally; pectoral fins dusky with irregular dark and pale bars. Pelvic fins mostly white, pink or pale orange basally. Dorsal fin dusky to black along distal margins of all membranes; first dorsal fin speckled with yellow and black on membrane between first two spines; second and third dorsal fins diagonally striped with faint reddish or dusky markings, markings roughly in line with semi-bars, those on side of body. Anal fin dusky red. Caudal fin dusky.

Females with head reddish above, dark brown behind centre of eye and onto upper portion of opercle; ventral half of head pale cream below eye, with some black and orange spots. Iris red to reddish brown. Snout with brownish black line from anterior margin of eye onto upper lip. Body pale pink, sides of body with orange and red marks forming pairs of discontinuous semi-bars and blotches, from dorsum to below lateral midline, last half-pair forming triangular mark on caudal peduncle; a row of white spots along mid-body between each dark blotch. Pectoral-fin base with a white splotch at lower edge, which seems to extend form white marking on lower portion of opercle, and red and white marks above; fin rays with alternating white and black bars. Pelvic fin white. First dorsal fin as in males; second dorsal fin diagonally striped with red markings; third dorsal fin banded with dusky red and white oblique bands. Anal fin with dusky red blotches along base. Caudal fin dusky, melanophores concentrated along outlines of ray shafts, interspaced with two white, vertical bars.

#### Colouration in preservative.

Males with head and body dusky, except belly and area behind eye clear. Body dusky with irregular double bars. Pectoral fins dusky with clear blotches on upper and lower margins of base. Distal halves of first and second dorsal fins dusky, membrane between first two spines of first dorsal with dense melanophores; third dorsal fin irregularly banded. Anal and caudal fins dusky.

Females generally pale to dusky. Top of head, opercle and pectoral-fin base with scattered melanophores; small clusters of melanophores below eye and along sides of mouth; a dusky bar of melanophores extending from eye onto upper lip. Body with faint, barely discernible, irregular markings. First dorsal fin as in males; second dorsal fin with clusters of melanophores near distal margin. Pectoral, third dorsal and caudal fins banded, melanophores concentrated along margins of fin elements. Anal fin with blotches of melanophores basally.

#### Etymology.

The specific name, *williamsi*, is in honor of Jeffrey T. Williams, Smithsonian Institution, National Museum of Natural History, in recognition of his excellent research work on marine blenniiform fishes.

#### Distribution.

The samples were collected from rocky shore areas with sand channels at depths of 1-3 m, along the southern coast of Taiwan.

#### Remarks.

*Helcogramma williamsi* shares the pattern of dentary pores and the numbers of fin rays and lateral-line scales with three congeners: *Helcogramma capidata* Rosenblatt (1960), *Helcogramma alkamr* Holleman (2007), and *Helcogramma rharhabe* Holleman (2007).These four species, as well as others in the *Helcogramma obtusirostris* species group, also share a putative synapomorphy- a blue line running from the corner of the mouth onto the preopercle in mature males.

However, *Helcogramma williamsi* is distinguished from *Helcogramma capidata* by its lobate supraorbital cirrus vs. without supraorbital cirrus; upper jaw extending to a point below anterior half of eye vs. extending to a point below posterior half of eye; and moderately complex cephalic sensory canal pores vs. rather simple pore pattern. *Helcogramma williamsi* can bedistinguished from *Helcogramma rharhabe* by the following features: vertebrae 10+26 vs. 10+24-25; males with yellow mark from anterior tip of upper lip to anterior margin of eye vs. crimson marks on upper lip on either side of center, black in the centre; body with 5-6 pairs of indistinct semi-bars vs. body of males almost entirely black with 3-4 pale narrow streaks from dorsum to midline. *Helcogramma williamsi* seems to be more similar to *Helcogramma alkamr* than to any other congeneric species in overall pattern of colouration. However, it can be distinguished from *Helcogramma alkamr* by the following features: height of first dorsal fin more than half height of second dorsal fin vs. height of first dorsal fin less than half height of second dorsal fin; lateral-line scales extending to a point below insertion between 2^nd^-4^th^ rays of third dorsal fin vs. lateral-line scales extending to a point just below the junction of second and third dorsal fins; ventral side of caudal peduncle with cycloid scales vs. ventral side naked; iris orange-red with reddish brown ring vs. red and pale gold.

### Helcogramma fuscipectoris

(Fowler, 1946)

http://species-id.net/wiki/Helcogramma_fuscipectoris

Fig. 3a

Enneapterygius fuscipectoris Fowler 1946: 186 (Type locality: Aguni shima, Riu kiu Island).
Enneapterygius personatus :Fowler 1946: 185.
Enneapterygius quadrimaculatus :Fowler 1946: 189.
Helcogramma obtusirostris (non Klunzinger 1871): Hansen 1986: 341 (part: Japan; Taiwan).
Helcogramma obtusirostris (non Klunzinger 1871): Shen and Wu 1994: 21.
Helcogramma fuscipectoris : Fricke 1997: 429.

#### Material Examined for Description.

NTOU-P 2009-06-058, male, 22.1 mm SL, Chenggong Township, Taitung County, Taiwan, intertidal rock pools, J. H. Huang, 6 June 2006; NTOU-P 2009-06-059, male, 22.3 mm SL, Chenggong Township, Taitung County, Taiwan, intertidal rock pools, M. C. Chiang and J. H. Huang, 19 Aug 2006; NTOU-P 2009-06-060, male, 22.0 mm SL, San-diao-jiao, Gongliao Township, Taipei County, Taiwan, J. T. Chen, 18 Aug 2006; NTOU-P 2009-06-61, male, 21.2 mm SL, Feng-chui-sha, Hengchun Township, Pingtung County, Taiwan, 6-12 m depth, M. C. Chiang and J. H. Huang., 20 July 2007; NTOU-P 2009-06-062, male, 21.8 mm SL, Ba-dou-zi, Keelung City, Taiwan, intertidal pools, M. C. Chiang and W. H. Li, 27 Aug 2008; NTOU-P 2009-06-063, male, 20.8 mm SL, Chenggong Township, Taitung County, Taiwan, intertidal pools, M. C. Chiang and J. H. Huang, 31 May 2008; NTOU-P 2009-06-064, male, 25.1 mm SL, Chenggong Township, Taitung County, Taiwan, intertidal pools, M. C. Chiang, 25 April 2009; NTOU-P 2009-06-065, 3 specimens, 20.0-23.3 mm SL, Da-bai-sha, Lyutao Township, Taitung County, Taiwan, 12 m depth, M. C. Chiang, 27 April 2009; NTOU-P 2009-06-066, female, 24.5 mm SL, Chenggong Township, Taitung County, Taiwan, intertidal pools, M. C. Chiang et al., 7 April 2007.

#### Description.

D III, XIII-XIV (modally XIV), 9-11 (modally 10). A I, 19-20. Lateral line with 21-22 pored scales (usually 22). Dentary pore pattern 3-4+1+3-4 (modally 4+1+4). Supraorbital cirrus simple and minute, sometimes too small to find. Nasal cirrus leaf-like and slender. First dorsal fin lower in height than second in both sexes. Vertebrae 10+26-27. Males with black mask on lower half of head below eye, black area extending onto base of pectoral-fin rays; fresh males with narrow, horizontal bright blue stripe extending from corner of mouth onto the preopercle, and a whitish blue dashed line on lower pectoral-fin base may be present. Fresh male specimens orange to red generally, series of pale marks and black or dark brown tiny dots along midline and back; females white or pale yellow with orange to red or brown markings extending from dorsum to midline or below, in which red to brown chromatophores are concentrated along lateral line. Dorsal-fin elements red. Anal fin with four, sometimes five or six, basal dusky red to black blotches. (Note. The orange/red body colouration described above is apparent after fresh specimens have been immersed in ice; when first captured, the head and body are pale olive to green or brownish green.)

**Figure 3.**
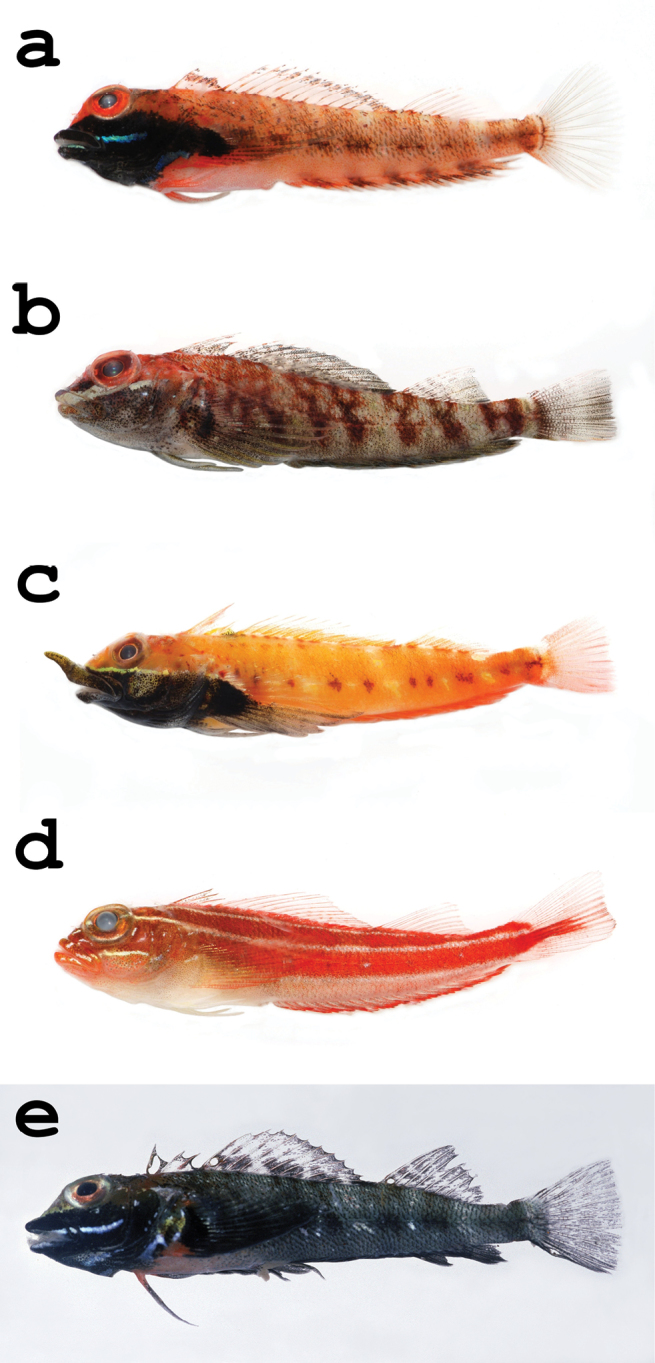
Specimen photographs of **a** *Helcogramma fuscipectoris*, NTOU-P 2009-06-063, male, 20.8 mm SL, Chenggong, Taitung, Taiwan **b** *Helcogramma inclinata*, NTOU-P 2009-06-054, male, 35.3 mm SL, Nan-ren-road, Pingtung, Taiwan **c** *Helcogramma rhinoceros*, NTOU-P 2009-06-043, 29.6 m SL, Feng-chui-sha, Pingtung, Taiwan **d** *Helcogramma striata*, NTOU-P 2009-06-048, 27.4 mm SL, Chuan-fan-shi, Pingtung, Taiwan **e** *Helcogramma trigloides*, male, 31.2 mm SL, Efate Island, Vanuatu, photograph by JT Williams.

#### Distribution.

The specimens described herein were collected at depths of 0-3 m from eastern and northeastern Taiwan. This species previously has been recorded from the eastern and southern coasts of Taiwan, Ryukyu Islands, Izu Islands (Japan), China, Philippines, Vietnam, Thailand, Malaysia, Indonesia, and Vanuatu (Fricke 1997).

#### Remarks.

*Helcogramma fuscipectoris*, *Enneapterygius personatus* Fowler (1946) and *Enneapterygius quadrimaculatus* Fowler (1946), which were described in the same paper, were subsequently placed in the synonymy of *Helcogramma obtusirostre* (Klunzinger 1871) by Hansen (1986). However, *Helcogramma fuscipectoris* was recognized by Fricke (1997) as a valid species, and he determined that *Enneapterygius personatus* and *Enneapterygius quadrimaculatus* were junior synonyms. *Helcogramma obtusirostre* is distinguished from *Helcogramma fuscipectoris* by geographical distribution, body colouration, anal fin colour pattern, and other characters (Fricke 1997).

### Helcogramma inclinata

(Fowler, 1946)

http://species-id.net/wiki/Helcogramma_inclinata

Fig. 3b

Enneapterygius inclinatus Fowler 1946: 190 (Type locality: Aguni shima, Riu Kiu Island).
Helcogramma habena Williams and McCormick 1990: 1026 (Type locality: Philippines)
Helcogramma habena : Shen and Wu 1994: 19.
Helcogramma inclinatum : Fricke 1997: 446.
Helcogramma inclinata : Williams and Howe 2003: 164.

#### Material Examined for Description.

NTOU-P 2009-06-050, 1 specimen, 29.0 mm SL, Chenggong Township, Taitung County, Taiwan, 6 m depth, J. H. Huang, 12 June 2006; NTOU-P 2009-06-051, 2 specimens, 30.6 and 32.2 mm SL, Chenggong Township, Taitung County, Taiwan, 6-9 m depth, M. C. Chiang and I-S. Chen, 19 Aug 2006; NTOU-P 2009-06-052, 1 specimen, 37.5 mm SL, Ma-gang, Taipei County, Taiwan, 5-8 m depth, M. C. Chiang et al., 1 Sep 2006; NTOU-P 2009-06-053, 2 specimens, 33.4 and 36.3 mm SL, NW shore of Liouciou Township, Pingtung County, Taiwan, 5-12 m depth, M. C. Chiang et al., 8 July 2007; NTOU-P 2009-06-054, 2 specimens , 33.4 and 35.3 mm SL, Nan-ren-road Ferry, Pingtung County, Taiwan, 5-10 m depth, M. C. Chiang et al., 19 July 2007; NTOU-P 2009-06-055, 1 specimen, 34.2 mm SL, Feng-chui-sha, Hengchun Township, Pingtung County, Taiwan, 1-3 m depth, M. C. Chiang et al., 3 June 2008; NTOU-P 2009-06-056, 2 specimens, 30.2 and 34.3 mm SL, Xian-jiao-wan, Pingtung County, Taiwan, 9 m depth, M. C. Chiang and W. H. Li, 8 Sep 2008; NTOU-P 2009-06-057, 2 specimens, 29.0 and 30.9 mm SL, Wan-Li-Tong, Pingtung County, Taiwan, 6 m depth, M. C. Chiang and W. H. Li, 10 Sep 2008.

#### Description.

D III, XIV-XV (modally XV), 10-11. A I, 20-22 (usually 21-22). Lateral line with 25-32 pored scales. Dentary pore pattern 7-10+5-7+7-11 (modally 8+6+8). Supraorbital cirrus small and pointed. Nasal cirrus simple and slender. First dorsal-fin height almost equal to second dorsal-fin height. Nape scales present. Vertebrae 10+28-29. Head mottled red to reddish brown, a white or blue line extending from tip of the upper jaw to dorsal angle of the preopercle; males with dark brown or black mask on lower half of head and the the blusish white line beneath eye conspicuous. Body with 7-8 reddish brown to brown oblique single bars or Y-shaped markings; males with more densely scattered melanophores over body. Dorsal fin with alternating white and reddish brown bands on spines and rays, many tiny melanophores speckled on membrane especially near the basal and marginal parts of fins. Anal fin gray or yellowish brown to black. Caudal fin translucent with dusky area basally and distally on center of fin.

#### Distribution.

This species has been recorded from the northeastern, eastern and southern shores of Taiwan, Ryukyu Islands, and the northern Philippines (Fricke 1997; Williams and Howe 2003).

#### Remarks.

*Helcogramma inclinata* was regarded as a junior synonym of *Helcogramma hudsoni* (Jordan and Seale 1906) by Hansen (1986). However, it had been recognised as a valid species and a senior synonym of *Helcogramma habena* (Williams & McCormick, 1990) by Fricke (1997) and Williams and Howe (2003).

### Helcogramma rhinoceros

Hansen, 1986

http://species-id.net/wiki/Helcogramma_rhinoceros

Fig. 3c

Helcogramma rhinoceros Hansen 1986: 344 (Type locality: Putic Island, Philippines).
Helcogramma rhinoceros : Fricke 1997: 467.

#### Material Examined for Description.

USNM 222370, holotype, 27.5 mm SL, N.W. Putic Island, Palawan province, Philippines, V. G. Springer, 22 May 1978; NTOU-P 2009-06-043, 1 male, 29.6 m SL, Feng-chui-sha, Hengchun Township, Pingtung County, Taiwan, 9 m depth, M. C. Chiang et al., 20 July 2007.

#### Description.

D III, XIV-XV, 10-11. A I, 20. Lateral line with 19-22 pored scales. Dentary pore pattern 4+1+4. Supraorbital cirrus small and pointed. Nasal cirrus simple and slender. First dorsal-fin height equal to second dorsal-fin height. Vertebrae 11+26. Males with a proboscis-like dermal prolongation on tip of upper lip. Head of males orange above; head below level of eye, including upper lip and its extension, black; black pigment extending onto basal portion of pectoral fin. A yellowish or bluish white line extending along edge of black mask from upper rim of upper jaw to opercle and onto pectoral-fin base. Body pale yellow with indistinct H-shaped yellowish orange to orange markings. In males, H-shaped markings diffuse, narrow pale saddle marks extending discontinuously from dorsum to midline and below. A row of reddish blotches present along lateral midline, yellowish orange spots present at dorsal-fin base, and a dark blotch comprising densely packed melanophores present at posterior base of first dorsal fin and at anterior base of second dorsal fin. Dorsal-fin spines and rays orange near distal margin of fin; membranes yellowish orange on basal half and spotted with small melanophores on distal half. Uppermost pectoral-fin rays translucent, lowermost grayish or blackish. Anal fin orange. Caudal fin pale red and semi-translucent. Colour pattern of females based on Hansen (1986): head and body overall lighter than males; body with same pigment pattern as males; head without dark mask but with irregular scattered melanophores on face and diffuse band from eye onto upper lip.

#### Distribution.

One specimen was collected in this study at a depth of 9 m from southern Taiwan. This species previously has been recorded from the Philippines, Thailand, Indonesia, Solomon Islands, and Vanuatu (Hansen 1986; Fricke 1997).

#### Remarks.

This species is recorded herein for the first time in Taiwanese waters.

### Helcogramma striata

Hansen, 1986

http://species-id.net/wiki/Helcogramma_striata

Fig. 3d

Helcogramma striata Hansen 1986: 349 (Type locality: Toga Point rocks, Miyakejima, Izu Islands, Japan).
Helcogramma striata :Shen and Wu 1994: 22.
Helcogramma striatum :Fricke 1997: 480.
Helcogramma striata :Holleman 2007: 77.

#### Material Examined for Description.

USNM 221667, holotype, 41.0 mm SL, Toga Point Rocks, Miyakejima, Izu Island, Japan, 34°07'N, 139°30'E, 1-3 m depth, P. E. Hadley and L. Cuyvers, 10 July 1977; NTOU-P 2009-06-044, 1 specimen, 20.5 mm SL, Wan-li-tong, Pingtung County, Taiwan, 6-9 m depth, M. C. Chiang et al., 14 Sep 2006; NTOU-P 2009-06-045, 1 specimen, 22.1 mm SL, Ho-bi-hu, Pingtung County, Taiwan, 12 m depth, M. C. Chiang and J. H. Huang, 2 June 2008; NTOU-P 2009-06-046, 1 specimen, 26.2 mm SL, Shan-hai, Pingtung County, Taiwan, 6-10 m depth, M. C. Chiang and J. H. Huang, 16 July 2008; NTOU-P 2009-06-047, 3 specimens, 25.5-26.4 mm SL, Xian-jiao-wan, Pingtung County, Taiwan, 9 m depth, M. C. Chiang and W. H. Li, 8 Sep 2008; NTOU-P 2009-06-048, 1 specimen, 27.4 mm SL, Chuan-fan-shi, Pingtung County, Taiwan, 9 m depth, M. C. Chiang and W. H. Li, 8 Sep 2008; NTOU-P 2009-06-049, 1 specimen, 21.6 mm SL, Hong-chai-keng, Pingtung County, Taiwan, 6-8 m depth, M. C. Chiang et al., 9 Sep 2008; NTOUP 201202-007, 1 male, 28.2 mm SL, Hong-chai-keng, Pingtung County, Taiwan, 9 m depth, M. C. Chiang et al., 5 Sep 2009; NTOUP 201202-008, 1 specimen, 28.5 mm SL, Nan-wan, Pingtung County, Taiwan, 10-12 m depth, M. C. Chiang et al., 27 May 2010.

#### Description.

D III, XIV-XV (modally XIV), 10-11 (modally 11). A I, 20-21 (modally 21). Lateral line with 18-20 (modally 18) pored scales. Dentary pore pattern 3+2+3. Supraorbital cirrus absent. Nasal cirrus simple and slender. First dorsal fin about half height of second dorsal fin. Vertebrae 10+27-28. Males and females with similar colour pattern. A characteristic colour pattern of three bluish white stripes along side of body: dorsalmost stripe originating over top of eye, extending backward along bases of dorsal fins; middle stripe originating on snout, extending through eye and terminating at caudal-fin base; ventralmost stripe originating on lips, extending across cheek and pectoral-fin base, and ending on ventral part of caudal peduncle. Dorsal part of head and body above ventralmost stripe red; body below ventral stripe gray or pink to white. Body with row of bluish or grayish white spots between middle and ventral stripes. Dorsal-fin elements and basal membranes reddish. Anal fin red. Caudal fin with red extending from body onto middle portion of fin.

#### Distribution.

The specimens described herein were collected at depths of 6-12 m from southern Taiwan. This species has been recorded in shallow waters of 1-20 m depth from the southern tip of Taiwan, Ryukyu Islands, Japan, Philippines, Sri Lanka, Australia, Thailand, Indonesia, Timor Sea, Papua New Guinea, Solomon Islands, Vanuatu, Fiji, Kiribati, and Line Islands (Fricke 1997).

### Helcogramma trigloides

(Bleeker, 1858)

http://species-id.net/wiki/Helcogramma_trigloides

Fig. 3e

Tripterygion trigloides Bleeker 1858: 234 (Type locality: Biliton Occidentalis, Indonesia).
Helcogramma trigloides : Fricke 1997: 489 (Neotype locality: Port Narevin, Erromango Island, Vanuatu).
Helcogramma trigloides : Hansen 1986: 351.

#### Material Examined for Description.

USNM 343890, Neotype, 33.3 mm SL, Port Narevin, Erromange Island, Vanuatu, 0-6 m depth, J. T. Williams et al., 28 May 1996.

#### Description.

D III, XIII, 9. A I, 18. Lateral line with 24 pored scales. Dentary pore pattern 3+3+3. Supraorbital cirrus small and semi-rounded. Nasal cirrus slender. First dorsal fin lower in height than second. Based on colour photo from Efate Island, Vanuatu by Jeffrey T. Williams. Body blackish green with indistinct black bars. Males with black mask on lower half of head below eye and a bluish white line extending from corner of mouth onto preopercle. Pectoral-fin base with yellow splotch and bluish white marks; a red blotch on lower base of pectoral fin and bases of ventralmost few rays. All fins dusky to black.

#### Distribution.

Although there are no specimens of this species in Taiwanese museums or other institutions, it has been recorded from the eastern shore of Taiwan (Fricke, 1997) and from Malaysia, Thailand, Indonesia, Palau New Guinea, Solomon Islands, and Vanuatu (Hansen 1986; Fricke 1997).

## Discussion

Within the genus *Helcogramma*, *Helcogramma williamsi* n. sp. and the *Helcogramma steinitzi* species group share the presence of dense micromelanophores on the membrane between the first two dorsal-fin spines in both males and females. However, *Helcogramma williamsi* is different from the *Helcogramma steinitzi* species group in having the distance between the first two dorsal-fin spines more than 1/2 of the distance between the second and third spines (vs. the distance between the first two dorsal-fin spines less than 1/2 of the distance between the second and third spines), the origin of the first dorsal behind a vertical through the posterior margin of the preopercle (vs. the origin of the first dorsal over the posterior margin of the preopercle), and the supraoccipital sensory canal forms a flattened curve (Fig. 2) anterior to the first dorsal-fin spines (vs. an open ‘V’-shaped anterior to the first dorsal-fin spines).

The common diagnostic feature of the *Helcogramma obtusirostre* species group is the blue line extending from the corner of the mouth onto the preopercle. *Helcogramma capidata*, *Helcogramma ellioti*, *Helcogramma fuscipectoris*, *Helcogramma obtusirostre*, *Helcogramma rharhabe*, *Helcogramma trigloides*, and *Helcogramma alkamr* share this character (Holleman, 2007) and thus may belong tothis species complex. The presence of a pale blue line extending from the corner of the mouth onto the preopercle in mature males of *Helcogramma williamsi* suggests that it is a member of the *Helcogramma obtusirostre* species group. However, further investigation is required to confirm this. More characters need to be proposed to distinguish the *Helcogramma obtusirostre* species group from other groups.

### Key to the species of *Helcogramma* from Taiwan:

**Table:** 

1	Trunk with several longitudinal stripes	*Helcogramma striata*
–	Trunk without longitudinal stripes	2
2	Symphyseal dentary pores 3 or more	3
–	Symphyseal dentary pores 1	4
3	Lateral line with more than 25 scales; anal fin with more than 20 rays; male with bluish white stripe from middle of upper lip to the dorsal angle of the preopercle	*Helcogramma inclinata*
–	Lateral line with fewer than 25 scales; anal fin with fewer than 19 rays; male with bluish white stripe from corner of jaw onto preopercle	*Helcogramma trigloides*
4	Upper lip with proboscis-like extension on males, head with horizontal yellowish or bluish white line from the upper rim of upper jaw to opercle	*Helcogramma rhinoceros*
–	Upper lip without proboscis-like extension; males with whitish blue line form corner of mouth to preopercle	5
5	Pattern of dentary pores 5+1+5	*Helcogramma williamsi* sp. n.
–	Pattern of dentary pores 4+1+4	*Helcogramma fusipectoris*

## Supplementary Material

XML Treatment for Helcogramma williamsi

XML Treatment for Helcogramma fuscipectoris

XML Treatment for Helcogramma inclinata

XML Treatment for Helcogramma rhinoceros

XML Treatment for Helcogramma striata

XML Treatment for Helcogramma trigloides
